# Immune System Disorder and Cancer-Associated Cachexia

**DOI:** 10.3390/cancers16091709

**Published:** 2024-04-27

**Authors:** Lingbing Zhang, Philip D. Bonomi

**Affiliations:** 1 Yinuoke Ltd., Changchun 130012, China; 2 Division of Hematology/Oncology, Rush University Medical Center, Chicago, IL 60612, USA; pbonomi@rush.edu

**Keywords:** CAC, cytokines, immune disorder, inflammation, muscle wasting, IL-6, TNF-α

## Abstract

**Simple Summary:**

Cancer-associated cachexia (CAC) is a serious condition wherein people with cancer lose muscle and fat, even with proper nutrition. It leads to weakness and can make treatment less effective. This review looks at how the body’s immune system and certain proteins affect CAC. Studies show that specific proteins, like IL-6 and TNF-α, play a crucial role in causing muscle and fat loss. Other cells in the immune system, called MDSCs, make CAC worse by causing inflammation. Clinical studies also found high levels of certain proteins in people with CAC. Understanding these processes might improve treatments, especially for those undergoing immunotherapy. A new drug called R-ketorolac has shown promise in reversing weight loss in animals. Combining drugs like this with immunotherapy could help patients with cancer suffering from CAC. However, more research is needed to fully understand how immune problems and CAC interact during treatment.

**Abstract:**

Cancer-associated cachexia (CAC) is a debilitating condition marked by muscle and fat loss, that is unresponsive to nutritional support and contributes significantly to morbidity and mortality in patients with cancer. Immune dysfunction, driven by cytokine imbalance, contributes to CAC progression. This review explores the potential relationship between CAC and anti-cancer immune response in pre-clinical and clinical studies. Pre-clinical studies showcase the involvement of cytokines like IL-1β, IL-6, IL-8, IFN-γ, TNF-α, and TGF-β, in CAC. IL-6 and TNF-α, interacting with muscle and adipose tissues, induce wasting through JAK/STAT and NF-κB pathways. Myeloid-derived suppressor cells (MDSCs) exacerbate CAC by promoting inflammation. Clinical studies confirm elevated pro-inflammatory cytokines (IL-6, IL-8, TNFα) and immune markers like the neutrophil-to-lymphocyte ratio (NLR) in patients with CAC. Thus, immunomodulatory mechanisms involved in CAC may impact the anti-neoplastic immune response. Inhibiting CAC mechanisms could enhance anti-cancer therapies, notably immunotherapy. R-ketorolac, a new immunomodulator, reversed the weight loss and increased survival in mice. Combining these agents with immunotherapy may benefit patients with cancer experiencing CAC. Further research is vital to understand the complex interplay between tumor-induced immune dysregulation and CAC during immunotherapy.

## 1. Introduction

Cancer-associated cachexia (CAC) is the persistent loss of skeletal muscle mass, with or without fat loss, that is not reversed by nutritional support [1]. In CAC, immune dysfunction caused by inflammation leads to a cytokine storm, raising the risk of multiple organ failure, and eventually impacting muscle and adipose tissue [2,3]. The immune system is implicated in CAC, considering immune disorder as a primary driver of CAC [3]. Clinically, it manifests as appetite loss, fatigue, weakness, and anemia leading to impaired functionality, low quality of life, unresponsiveness to treatment, and early mortality in advanced stages [3,4,5]. The incidence of CAC is particularly high in late-stage cancer, with 60–80% of patients experiencing it [6]. CAC is more prevalent in pancreatic, gastro-esophageal, and head and neck cancers [4]. Globally, approximately 8.2 million deaths annually are attributed to cancers, with CAC playing a significant role in most cases [4].

Cancer is characterized by immune dysfunction and inflammation [7]. Consequently, immune tolerance is a vital characteristic of cancer cells, and its impairment can lead to autoimmunity [8]. In late-stage cancer and autoimmune disorders, elevated cytokine levels and autoantibodies targeting multiple organs are produced [9]. The upregulated cytokines within the tumor microenvironment interact with muscle cells causing muscle wasting [6]. Several cytokines such as IL-1β, IL-6, and TNF-α have been implicated in CAC [10,11,12]. In patients with colorectal CAC, tumors exhibited elevated inflammatory cytokines (IFN-α and IL-8), growth factors (granulocyte-macrophage colony-stimulating factor and epidermal growth factor), actin, and collagen deposits [13]. The dysregulation of IFN-γ was shown in the white adipose tissue (WAT) of cachectic rats [14]. Evidence suggests that muscle wasting induced by IFN-γ or TNF-α involves the NF-κB pathway [12]. Immune cells can mediate inflammatory responses associated with CAC, as demonstrated by the reduction in muscle weight loss in diabetic/prediabetic mice through the infusion of CD4+CD44v.low naïve T-cells [15].

CAC presents a complex syndrome involving alterations in several metabolic pathways and the immune system, making the identification of effective therapeutic targets challenging. CAC-related complications hinder clinical evaluation as the understanding of immune cell roles, responses to altered nutrients and tissue inflammation remains unclear [1]. Clinical data are scarce as CAC predominantly occurs in late-stage cancer, when conducting invasive metabolic tests and biopsies is difficult, resulting in a reduced patient turnout. Consequently, mechanistic studies have primarily been conducted in mice, although disparities exist between these models and clinical findings [4]. This review seeks to explore pre-clinical and clinical evidence linking tumor-induced immune disorder to CAC, thereby establishing the causative role of tumor-induced immune disorders in CAC development. Additionally, it emphasizes the potential treatment approach of simultaneously suppressing multiple inflammatory cytokines to alleviate CAC.

## 2. Methodology

**Figure 1:**
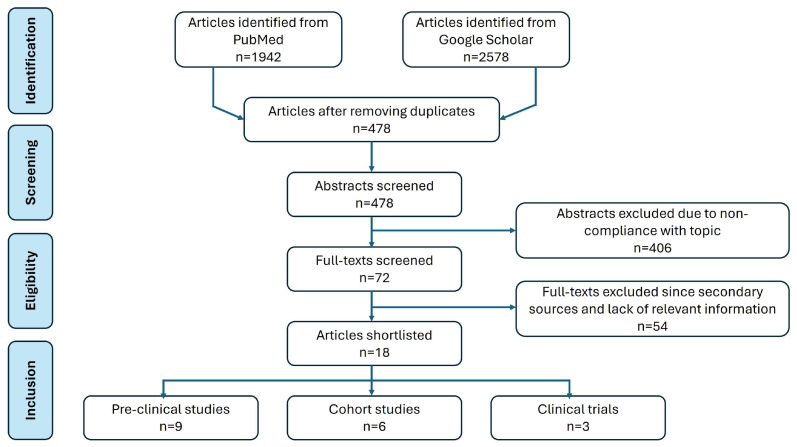
Flowchart of literature search and article screening.

Scholarly papers published in English from January 2000 to January 2023 were searched using PubMed and Google Scholar databases with combinations of the appropriate keywords, medical subject headings (MeSH), and suitable Boolean operators. The search terms employed were “cancer cachexia”, “inflammation”, “cytokine”, and “immune disorder”. All published original articles, comprising pre-clinical and clinical studies, were considered. Studies beyond 10 years were excluded from the search. Additionally, studies not reporting CAC, inflammation, and anti-inflammatory interventions were not considered. After eliminating the duplicates, the titles and abstracts were assessed and publications with full texts that met the inclusion criteria were further evaluated (Figure 1).

## 3. Pre-Clinical Evidence of Tumor-Induced Immune Disorder Driving CAC

**Figure 2:**
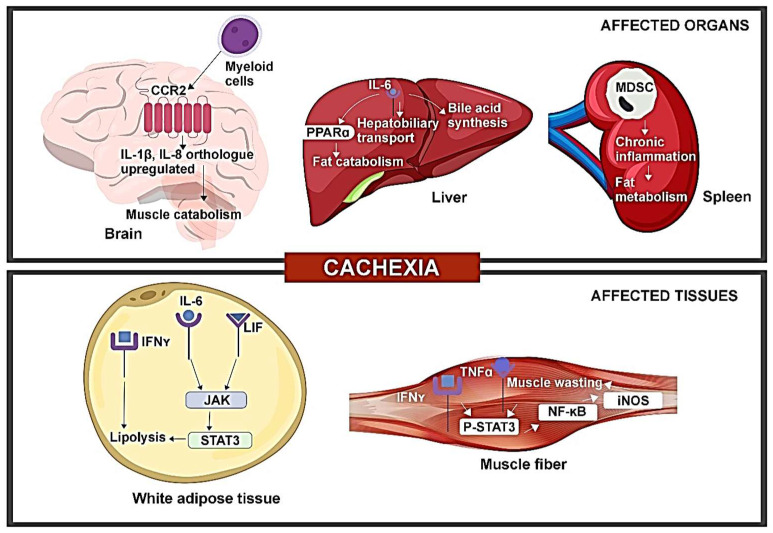
Tumor-induced immune disorder drives CAC development in organs and tissues.

CAC has been linked to elevation or alterations in inflammatory cytokines through various mouse models, offering mechanistic insight into the impact of cytokines on CAC (Figure 2).

Alterations in serum IL-6 and leptin levels in CAC are influenced by leukemia inhibitory factor (LIF) through the JAK-dependent pathway. A study involving colon adenocarcinoma (C26c20) and recombinant LIF-induced CAC mouse models revealed elevated serum LIF and IL-6 levels [16]. Additionally, both CAC mouse models exhibited a reduced leptin expression in WAT. IL-6 and LIF were both capable of inducing lipolysis, with LIF stimulating lipolysis at a lower concentration, while IL-6 resulted in a higher maximum lipolysis. Both cytokines could activate the JAK/STAT pathway. JAK inhibitors like tofacitinib and ruxolitinib markedly inhibited IL-6-mediated lipolysis in adipocytes. Treatment with ruxolitinib reduced adipose loss and anorexia in both mouse models. The findings show that multiple molecules are associated with CAC, suggesting that targeting individual molecules may not reverse CAC or impact its immunomodulatory effects. Targeting common pathways like JAK can suppress the phenotype of the CAC pathway, although the simultaneous effect of the STAT3 feedback regulator on JAK inhibition requires further investigation [16].

IL-6 produced from tumors can impact the potential of the liver to respond to reduced calorie intake. In mouse models of colorectal cancer (C26 model) and pancreatic ductal adenocarcinoma (PDAC) (LSL-Kras^G12D/+^, LSL-Trp53^R17H/+^, Pdx-1-Cre/+ (KPC) model), reduced body weight, muscle mass depletion, and elevated catabolism markers were seen in muscles for both CAC models. Pre-cachectic mice showed a decreased expression of PPARα, a transcription factor of ketogenesis, and its target genes *Acadm* and *Hmgcs2*, suppressed by IL-6 in a dose-dependent manner. Metabolic stress indicators included lowered fasting glucose and elevated fasting corticosterone levels. Transcriptomic analysis of C26 tumors (both cachectic and pre-cachectic) revealed downregulated immune pathways, affecting NK cells, CD3+T cells, CD8+T cells, and CD4+Foxp3-T cells. Elevated corticosterone levels may reduce the effectiveness of immunotherapy in controlling cancer progression [17]. This is consistent with the clinical observation that CAC is prognostic of poor response to cancer immunotherapy [18,19].

The increased expression of inflammatory cytokines—IL-1β, 1L-8 orthologues was observed in the brain of the PDAC mouse model. The PDAC mice, when injected with C57BL/6 Kras^G12D^ Tp53^R172H^ Pdx1-Cre^+/+^ (KPC) cells, showed increased levels of IL-1β, IL-1R in the hypothalamus and postrema, while expression of anti-inflammatory IL-10 was increased in postrema. Additionally, increased levels of IL-8 orthologues, Cxcl1 and Cxcl2, were also seen. The administration of a CCR2 inhibitor resulted in reduced CD45+ globoid cells in VI meninges, decreased anorexia, and muscle catabolism, indicating that inhibiting CCR2 could reduce immune cell infiltration and CAC. Furthermore, the exclusive expression of CCL2 and infiltration of CCR2+ immune cells were found in the VI meninges of mice with PDAC. Blocking P2RX7 (purinergic receptor of macrophages) prevented the infiltration of neutrophils and CD45^high^ myeloid cells, suggesting a role for CCR2/CCL2 in mediating CAC. While multiple factors contribute to PDAC-associated CAC, targeting neutrophils or CCR/CCL2 may reduce CAC symptoms partially [11].

Alterations in IFN-γ signaling were identified during the progression of CAC. A study on WAT of Walker-256 rodents found anorexia and elevated levels of inflammatory cytokines such as TNFα, IL-1β, and IL-6. The 14-day progression of CAC post-tumor injection correlated with increased tumor weight and significantly reduced calorie intake on the 14th day. Metabolic changes included elevated triglyceride and non-esterified fatty acids, along with decreased serum glucose and protein concentration. Mesenteric pads exhibited an increased expression of IFN-γ, *Ifng1* gene (IFN-γ receptor subunit 1), and *Ifng2* gene (IFN-γ receptor subunit 2), while retroperitoneal pads showed a reduced expression of IFN-γ and *Ifng1* gene with unchanged *Ifng2* gene expression. IFN-γ levels were significantly correlated with the lipolysis of WAT, as evidenced by the increased free fatty acid (FFA) concentration in late-stage CAC. These findings indicate dysregulated IFN-γ signaling on a depot-specific basis in cachectic mice, contributing to immune disorder in CAC [14]. Furthermore, reports suggest that chronic inflammation induced by cytokines and myokines leads to the browning of WAT in animal models, which is associated with CAC. This transformation of white adipocytes into brown adipocytes contributes to lipid mobilization and increases energy expenditure, highlighting an early aspect of CAC pathophysiology [20].

TNFα/IFN-γ induces CAC through the STAT3-NF-κB complex. In a study involving C56BL/6J wild-type, IL-6 knockout (KO), and inducible nitric oxide synthase KO mice, TNFα/IFN-γ led to a marked reduction in muscle fibers and increased pγ-STAT3 levels in C2C12 myotubes. Consequently, the effector of IL-6-induced muscle loss, STAT3, was also activated by TNFα/IFN-γ. Enhanced pγ-STAT3 levels were found in the gastrocnemius muscle of wild-type and IL-6 KO mice, but muscle wasting mediated by TNFα/IFN-γ was absent in the latter, demonstrating that STAT3 activation by TNFα/IFN-γ does not involve IL-6. Additionally, JAK inhibitors prevented the TNFα/IFN-γ mediated muscle loss and STAT3 phosphorylation. Treatment with TNFα/IFN-γ caused the increased nuclear translocation of pγ-STAT3, leading to a significant elevation of iNOS expression, as regulated by the binding of pγ-STAT3 and p65 to the *iNos* promoter in cachectic muscles. The study also revealed an interaction between STAT3 and the p65 subunit of NF-κB, indicating that the nuclear translocation of pγ-STAT3 and iNOS expression is dependent on NF-κB. Thus, the results indicated that cytokines like IL-6 or TNFα/IFN-γ can independently activate STAT3, thereby inducing CAC [12].

The expansion of myeloid-derived suppressor cells (MDSCs) appears to promote the development of CAC. The characterization of immune cells in the brain of PDAC mice revealed augmented CD45^high^CD11b+myeloid cells (mostly neutrophils), Ly6C^high^ monocytes, and Ly6C^low^ myeloid cells, indicating immune cell infiltration in the brain. Notably, microglia in the CNS parenchyma phagocytized infiltrating neutrophils, particularly in the thalamus and cortex, highlighting the protective role of CNS parenchyma during inflammation [11]. A study involving Balb/c and C57BL/6J female mice showed that MDSC expansion and tumor burden are linked to decreased adipose tissue and an increased hepatic concentration of acute phase proteins and oxygen consumption in 4T1 mice as compared to 66C4 bearing mice, indicating that the expansion could accelerate CAC [21]. The study also reported an increase in the number of Gr-1^+^CD11b^+^ splenocytes, mainly comprising MHC-IIdim/− (90%), and a smaller number of CD31+ (5%) in 4T1 mice with tumor growth. These expanded Gr-1^+^CD11b^+^ cells produced high levels of reactive oxygen species (ROS) and suppressed the proliferation of T cells. Late-stage 4T1 mice were found to be susceptible to infection, leading to increased mortality due to pulmonary infiltration and liver necrosis. The authors suggested that MDSCs in 4T1-bearing mice causes inflammation and plays a pivotal role in CAC. Therefore, anti-cancer therapies targeting the immunosuppression ability of MDSCs may prove effective against CAC [21].

Immune disorder hinders hepatobiliary transport in CAC. A transcriptome-based study using C26 mice identified two down-regulated genes, *Ugt2b1* and *Oatp2*, encoding a drug-metabolizing enzyme and a bile transporter, respectively [22]. Elevated levels of bilirubin, bile acids, and their conjugated form, along with a reduced expression of genes related to bile acid synthesis, indicated altered bile acid enterohepatic cycle and hepatobiliary transport during CAC progression. Taurine synthesis and taurine transporter genes were also downregulated. The liver of CAC mice showed a high abundance of NF-κB-p65 complex, and transcriptomic analysis revealed increased expressions of IL-Iβ and TNFα. IL-6 regulated genes related to hepatobiliary transport and a positive correlation was observed between the bile acid levels and IL-6, C-reactive protein, and weight loss. The liver of CAC mice exhibited an abundance of neutrophils, suggesting the development of cholestasis in CAC [22].

Despite extensive pre-clinical evidence indicating that tumor-induced immune disorder drives the development of CAC, there is still a lack of evidence demonstrating that immune modulation can ameliorate CAC symptoms in cachectic mice. A recently published study involving C26 mice not only identified abnormalities in immune cells and underlying inflammatory cytokines but also found that treatment with R-ketorolac, an immunomodulator, mitigated the weight loss independent of nutrition intake and tumor growth, alleviating CAC symptoms and increased murine survival rate. In addition, an increased lymphocyte count and reduced IL-6 blood levels in the R-ketorolac treated cohort provide evidence of the immune modulatory effects of this agent. R-ketorolac’s reversal of CAC and its immune modulatory effects appear to be independent of COX inhibition. Significantly, in the C26 mice model, a combined treatment of R-ketorolac and chemotherapy resulted in a 100% survival rate. These pre-clinical observations highlight the therapeutic potential of immunomodulators in treating CAC [23].

## 4. Cellular and Molecular Immunomodulation Associated with CAC in Patients with Late-Stage Cancer

Clinical investigations have further validated pre-clinical findings to identify suitable biomarkers for CAC prognosis or characterize cytokine expression differences based on gender and cancer type. Immune disorder was seen to be associated with CAC, using the NLR as a biomarker of immune dysfunction. A multicenter cohort study involving patients (n = 2612) with CAC assessed the relationship between NLR and overall survival. NLR was significantly elevated in patients experiencing CAC with pancreatic, colorectal, cervical, liver, and lung cancer. Patients with late-stage CAC exhibited higher NLR levels than patients with non-metastatic stage cancer. The dose–response association between NLR and overall survival revealed an L-shaped curve with a threshold of 3.5. High NLR was linked to poor prognoses, male sex, old age, metastatic disease, increased bilirubin and platelets, decreased RBC, and reduced food intake. Symptoms of CAC such as anorexia, lower food intake, and a decline in the quality of life were observed in patients with high NLR. These findings suggest the potential utility of including NLR in diagnostic assessments for CAC prognosis and treatment approaches [23,24]. This study establishes a correlation between immune disorder and survival in CAC.

A study involving patients with cachectic gastrointestinal cancer (n = 38; 21 CAC and 17 weight stable cancer (WSC) identified an altered TGFβ signaling pathway in patients with CAC, reduced levels of hemoglobin and albumin, and elevated C-reactive protein, IL6, and TNFα, an indicating immune disorder [25]. Morphological changes in adipose tissue included alterations in adipocyte shape, collagen deposits, mature elastic fibers, collagen accumulation, increased collagen type I, III, VI, fibronectin expression, α-smooth muscle actin, and fibroblast-specific protein, indicating activated myoblasts. Furthermore, elevated levels of TGFβ1, TGFβ3, and its effector molecule Smad4 confirmed the upregulation of the TGFβ pathway in the adipose tissue of patients having CAC [25].

Increased serum IL-8 levels were associated with CAC and sarcopenia in patients with pancreatic cancer, as evidenced by a study involving resected (n = 91) and locally advanced (LA) groups (n = 55). Among the 110 identified patients having CAC (resected: 59, LA: 51) with significantly low survival, IL-8 levels were elevated in the resected group, correlating with anorexia, tumor size, and CA19-9 levels. CAC was found in most patients in the LA group and was associated with BMI, weight loss, anorexia, fatigue, and survival. Elevated IL-6 levels were observed in all CAC patients except the LA group. While in tumors, the increased IL-8 level was related to shorter disease-free survival, in serum, it was related to low overall and disease-free survival. Finally, increased IL-8 expression and CAC were found to be independent predictors of overall survival, and hence measuring serum IL-8 levels is suggested for evaluating the prognosis of patients with pancreatic cancer, while further studies are required to assess the therapeutic potential of IL-8 inhibition [26].

A study involving patients with cancer (n = 311) identified elevated IL-6 levels in those experiencing CAC (n = 74), accompanied by a significant increase in platelets, and FFA levels [10]. This was coupled with reduced total protein, albumin, cholesterol, hemoglobin, lymphocyte, and BMI. Gender-specific analysis revealed IL-6 elevation in both genders, with increased platelets in males and elevated FFA in females. Plasma IL-6 and FFA concentrations were positively correlated in female patients with CAC. Significant differences were observed between patients with gastric and colorectal cancer experiencing CAC, showing that increased IL-6 was found in patients with gastric cancer CAC and patients with colorectal cancer CAC exhibiting high IL-6, TNF-α, platelet, white blood cell count, FFA, and ApoA. This study suggested that gender-specific analysis should be conducted for patients with CAC, and further research is needed to understand the mechanisms related to abnormal fat metabolism [10].

The NLRP3 inflammasome pathway is activated in the adipose tissue of patients with CAC. A study conducted in patients having colorectal cancer (n = 20; 10 CAC and 10 WSC) found reduced low-density lipoprotein (LDL) and cholesterol levels in patients with CAC than in WSC. Elevated CRP levels and reduced hemoglobin levels were found in CAC compared to controls. Lipopolysaccharide (LPS) activation caused elevated p50 levels in the CAC of peritumoral adipose tissue (PtAT), while in subcutaneous explants (ScAT), p50 levels were suppressed by LPS. However, mean p50 levels were higher in ScAT, suggesting potential NLRP3 overexpression. Since the NF-κB p50/p65 complex induces the NLRP3 expression, higher levels of p50/p65 correlated with a higher NLRP3 expression. Elevated NLRP3 expression was observed in both CAC explants and the caspase 1 protein concentration was significantly increased in the ScAT of CAC and WSC. In ScAt explants, LPS caused elevated levels of IL-18 and its protein content. The expression of TLR4 showed depot-specific variation with elevated levels in PtAT and an unaltered expression in ScAT. LPS-induced TLR4 expression reversed depot-specific variation, and increased p65 levels in both explants were associated with the overexpression of IL-1β. Together, the results indicate that adipose tissue in CAC is involved in inflammation and its depot-specific regulation [27].

Clinical studies revealed the overexpression of cytokines such as IL-6 and IL-8 in patients with CAC. Changes in the TGFβ pathway and increased NLR were also observed with CAC progression. Furthermore, inflammasome activation was found in patients with CAC (Table 1).

**Figure 3:**
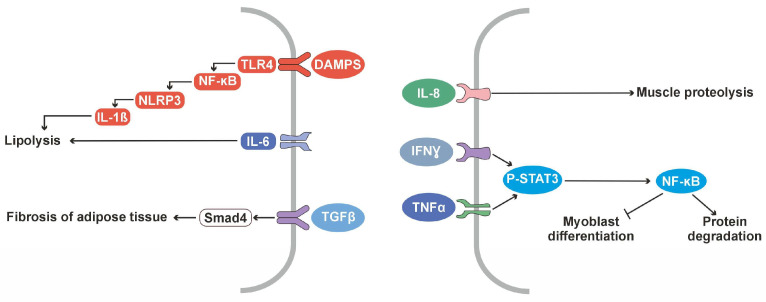
Major altered cytokines identified in CAC by clinical studies.

These clinical findings emphasize that inflammation contributes to CAC through various immune mechanisms (Figure 3), thus suggesting immune modulation as a potential therapeutic approach for addressing the complex syndrome.

## 5. Clinical Implications and Future Directions

Research displays a link between CAC, metabolic pathways, and tumor immunology. IL-6 was implicated in modulating ketogenesis in CAC mice, inducing metabolic stress and immune suppression mediated by glucocorticoids [17]. IFN-γ, an immune response effector, was found to be involved in the lipolysis of WAT [14]. Administering ruxolitinib led to a reduction in adipose loss and alleviated anorexia in CAC mouse models [16]. Inflammation-induced cholestasis was observed in CAC, and cholestyramine treatment reduced the expression of inflammatory pathway genes [22]. Apart from the upregulation of inflammatory factors, neutrophil infiltration was observed in the brain, aggravating CAC symptoms where the inhibition of CCR2 reduced neuroinflammation and attenuated CAC [11]. These findings suggest that immune modulation, whether caused by cancer cells, immune cells, or cytokines, induces metabolic alterations in CAC. R-ketorolac, an immunomodulator, reduced the weight loss in C26 and CHX207 mice by decreasing both adipose tissue and skeletal muscle loss. A dosage of 2 mg/kg/day of R-Ketorolac was sufficient to stop weight loss in tumor-bearing mice without affecting COX activity. This is particularly noteworthy since a major drawback of long-term NSAID use is the side effect of COX-1 inhibition [28]. Thus, R-ketorolac can alleviate CAC symptoms by modulating the immune system towards a near-normal state, preventing death in C26 mice [23]. Importantly, treating CAC through immunomodulation may improve the inferior response of patients with elevated inflammatory markers to checkpoint inhibitor immunotherapy [29,30,31]. Hence, mitigating CAC symptoms may be achievable by ameliorating immune disorders.

Several therapeutic strategies targeting the cytokines in CAC have been completed or are currently in clinical trials. In a randomized phase I trial (NCT00201812) involving patients with late-stage cancer (n = 36), treatment with etanercept, a TNF-α receptor decoy, and docetaxel reduced toxicity and fatigue. However, increased docetaxel concentration resulted in neutropenia and was associated with the upregulation of NF-κB and TNF-α [32]. Another randomized, blinded study with patients with non-small-cell lung cancer (NSCLC) (n = 124) showed effectiveness in reducing anemia and CAC after treatment with ALD518, an anti-IL-6 antibody [33]. In a phase II study (NCT01206335) involving patients with cancer (n = 21), treatment with OHR 118, an immunomodulator, improved the symptoms of CAC [34]. Recently, trials for new investigational interventions have been initiated in the clinical setting. An ongoing “proof of concept” (POC) trial, KetoROCX (NCT05336266), involving patients with PDAC, is aimed at gathering preliminary evidence demonstrating the feasibility and efficacy of ketorolac treatment in reducing CAC symptoms to prove the concept that CAC could be ameliorated through immunomodulation [35]. Exploring these promising avenues is crucial to advancing our understanding and developing effective treatments for CAC.

## 6. Strengths and Limitations of the Study

This study offers a robust and comprehensive review of CAC, synthesizing findings from pre-clinical and clinical studies to provide a thorough understanding of its pathophysiology and clinical implications. By integrating evidence from animal models and human patients, this study elucidates the complex interplay between immune dysfunction, inflammation, and metabolic pathways in driving CAC progression. This integrative approach enhances the depth of analysis and facilitates the identification of potential therapeutic targets for mitigating CAC symptoms. Additionally, this study’s emphasis on immune modulation as a promising therapeutic strategy highlights its relevance in current clinical practice and ongoing research efforts. By discussing the clinical implications of immune dysregulation in CAC and highlighting ongoing clinical trials investigating novel interventions, this study effectively translates pre-clinical insights into actionable strategies for improving patient outcomes.

Despite its strengths, this study has several limitations that warrant consideration. One notable limitation is the heavy reliance on animal models to elucidate the mechanisms of CAC, which may not fully capture the complexity of human disease pathogenesis and treatment responses. This reliance on animal data introduces a potential translational gap and underscores the need for the cautious interpretation of pre-clinical findings in the context of human disease. Furthermore, the heterogeneity observed in clinical studies included in this review, such as variability in patient populations and study designs, may introduce bias and limit the generalizability of the findings. Additionally, the lack of longitudinal data tracking CAC progression and treatment responses over time hinders the ability to accurately assess causality and long-term outcomes. Despite these limitations, this study provides valuable insights into the role of immune dysregulation in CAC and identifies avenues for future research to address these gaps in knowledge.

## 7. Conclusions

CAC is a complex, multifactorial syndrome involving metabolic and immune modulation. Accumulating evidence suggests the role of pro-inflammatory cytokines and immune disorders in driving CAC. The inhibition of cytokines and immunomodulation has shown promise in reducing CAC symptoms. Therefore, targeting cytokines or modulating the immune system may represent an effective therapeutic approach that reverses weight loss, palliates symptoms associated with CAC, and could conceivably enhance the effectiveness of immunotherapy regimens.

Currently, CAC research faces several challenges, including the unclear function of immune cells within a tissue, their response to inflammation and reduced caloric intake, the lack of well-developed mouse models mimicking CAC characteristics, and the absence of standard diagnostic methods for CAC identification. This lack of comprehensive understanding of CAC mechanisms hinders the development of effective treatment approaches. This review highlights the significance of immune disorder as a crucial mechanism in CAC and underscores a therapeutic approach through immunomodulation to reduce CAC and improve survival.

## Figures and Tables

**Table 1:** Summary of clinical studies highlighting the role of inflammation in CAC.

Study and Reference	Cancer Type	Sample Size	Objective	Outcome
Zhang et al., 2021 [24]	Solid malignant tumors	N = 2612	Evaluation of NLR as a biomarker in CAC	NLR increases in CAC NLR > 3.5 can predict survival
Alves et al., 2017 [25]	Gastrointestinal cancer	N = 59	Assessment of changes in extracellular matrix in CAC	Fibrosis of adipose tissue and alterations in TGFβ level observed
		CAC: 21		
		WSC: 17		
		Control: 21		
Hou et al., 2018 [26]	Pancreatic cancer	N = 146	Relation between CAC status and inflammatory cytokines	Serum IL-8 level can predict survival IL-8 can be an indicator of pancreatic cancer
		LA group: 55 (CAC: 51, Non-CAC: 4)		
		Resected group: 91 (CAC: 59, Non-CAC: 32)		
Han et al., 2019 [10]	Gastric cancer or colorectal cancer	N = 311	Clinical characteristics of patients (CAC and non-CAC) regarding the level of inflammation factors and lipid metabolism parameters	IL-6 levels varied between patient groups; females with CAC exhibited elevated IL-6 and FFA levels
		CAC: 74		
		Non-CAC: 237		
de Jesus et al., 2021 [27]	Colorectal tumors	N = 28 CAC: 10 WSC: 10 Control: 8	To confirm that NLRP3 inflammasome pathways are triggered in adipose tissue of CAC	Adipose tissue in CAC is involved in inflammation and its regulation is depot-specific

CAC: Cancer-associated cachexia; FFA: Free fatty acid; LA: Locally advanced; NLR: Neutrophil-to-lymphocyte ratio; WSC: Weight stable cancer.
